# Case report: Androgen-secreting adrenocortical tumors in eight cats

**DOI:** 10.3389/fvets.2023.1158142

**Published:** 2023-06-13

**Authors:** Caylen G. Erger, Allison L. Gerras, Alan J. Conley, Chen Gilor, Karah Burns DeMarle, Kent R. Refsal, Jamie M. Fleming, Dodd G. Sledge, Daniel K. Langlois

**Affiliations:** ^1^Department of Small Animal Clinical Sciences, College of Veterinary Medicine, Michigan State University, East Lansing, MI, United States; ^2^Department of Pathobiology and Diagnostic Investigation, College of Veterinary Medicine, Michigan State University, East Lansing, MI, United States; ^3^Department of Population Health & Reproduction, School of Veterinary Medicine, University of California, Davis, Davis, CA, United States; ^4^Department of Small Animal Clinical Sciences, College of Veterinary Medicine, University of Florida, Gainesville, FL, United States; ^5^Department of Veterinary Clinical Sciences, College of Veterinary Medicine, The Ohio State University, Columbus, OH, United States; ^6^Veterinary Diagnostic Laboratory, College of Veterinary Medicine, Michigan State University, Lansing, MI, United States; ^7^BluePearl Pet Hospital, Oak Creek, WI, United States

**Keywords:** adrenal, aggression, androstenedione, behavior, periuria, testosterone

## Abstract

Urine marking, aggression, and other behavioral concerns are common reasons for cat owners to seek veterinary care. Empiric treatment for lower urinary tract disease or primary behavior disorders are commonly pursued, especially in those cases with normal routine laboratory evaluations. Herein, we report the clinicopathologic findings in eight sexually altered cats that were diagnosed with androgen-secreting adrenocortical tumors. Nearly all cats (*n* = 7) initially were evaluated for inappropriate urination and pungent urine, with additional behavioral concerns including aggression (*n* = 3) and excess vocalization (*n* = 4) commonly reported. Penile barbs (*n* = 5) were identified in all five male cats, and an enlarged clitoris was observed in one female cat. Testing of serum androgen concentrations revealed abnormally high androstenedione (*n* = 1) or testosterone (*n* = 7) concentrations. In the five cases with available adrenal tissue, histopathologic evaluation identified either an adrenocortical adenoma (*n* = 3) or adrenocortical carcinoma (*n* = 2). Hormonal abnormalities resolved and clinical signs improved in the four cats that underwent surgical adrenalectomy, with each of these cats surviving >1 year. However, clinical signs were minimally impacted with medical treatments, including one cat in which trilostane treatment failed to improve clinical signs or testosterone concentrations. This collection of cases underscores the importance of a detailed physical examination as well as the consideration of endocrine disturbances in cats undergoing evaluation for inappropriate urination or aggression. Furthermore, this report adds to the growing body of evidence that sex-hormone secreting adrenal tumors in cats may be an under-recognized syndrome.

## 1. Introduction

Behavioral abnormalities are a common reason for pet owners to seek veterinary care, with inappropriate urination and aggression often cited as the most common behavior problems in cats (1–4). These disorders negatively impact the human-animal bond and can decrease the likelihood of the cat receiving appropriate veterinary care (5, 6). Inappropriate urination and aggression also are leading causes for pet abandonment as well as non-medical elective euthanasia (7–10).

Often, behavioral disorders in cats are treated empirically once routine laboratory tests (e.g., chemistry profiles, urinalyses) have been performed without identification of an underlying medical condition. This often includes environmental modifications or treatments with behavior-modifying medications (e.g., amitriptyline, fluoxetine). Yet ~ 10% of cats have inadequate treatment responses (11–15). Occult medical conditions might be contributing to behavioral problems in some of these cases as comprehensive diagnostic evaluations are seldom performed.

Aldosterone-secreting adrenal tumors are well-described in cats and are thought to be the most common form of corticosteroid-secreting adrenal tumor in this species (16). However, reports of sex-hormone secreting adrenal tumors are increasing in frequency in recent years (17–30). Most of studies describe hyperprogesteronism with secondary diabetes mellitus (17–25), with only seven cases of androgen-secreting adrenal tumors described (26–30). The primary objectives of this study were to describe the clinicopathologic features and management of 8 cats with androgen-secreting adrenal tumors, all of which were initially evaluated for behavioral concerns.

## 2. Case series description

### 2.1. History and physical examination

Eight neutered cats (five males, three females) from seven veterinary clinics spanning the years 2014–2022 were included in this study. Median (range) age and body weight were 8.5 years (7–15 years) and 4.7 kg (3.77–5.5 kg). Breeds included domestic short hair (*n* = 6), Maine Coon mix (*n* = 1), and domestic long hair (*n* = 1). All cats were evaluated for ongoing behavioral problems, including periuria or urine marking in seven cats (cases 1–7), increased vocalization in four cats (cases 1, 2, 4, and 6), and aggression in three cats (cases 2, 3, and 8). Other presenting complaints included malodorous urine (cases 1–7), weight loss (cases 1, 2, and 4), and increased appetite (case 2). Newly developed aggression toward other cats in the household was the only clinical sign reported in one cat (case 8).

Various treatments were attempted prior to documentation of increased androgen concentrations in cases 1–7. Five cats (cases 1, 2, 4, 5, and 7) were treated with antibiotics for potential urinary tract infection, but periuria did not resolve in any of these cases. Three cats (cases 2, 3, and 6) were treated with behavior-modifying medications for at least 1 month in duration, which included treatment with amitriptyline (cases 3 and 6), fluoxetine (case 2), or selegiline (case 3). Empiric treatments for possible feline idiopathic cystitis, including the use of Feliway^®^ diffusers (case 1), prescription urinary diets (cases 1 and 4), buprenorphine (case 4), and altering litter types (case 1), were also attempted in two cases. Behavioral and environmental modification did not resolve the inappropriate urination or aggression, although 1 cat (case 6) had mild and transient improvement of periuria. One cat (case 3) was treated with the progestin medroxyprogesterone acetate, which lessened the severity of malodorous urine but did not alter aggression.

**Figure 1:**
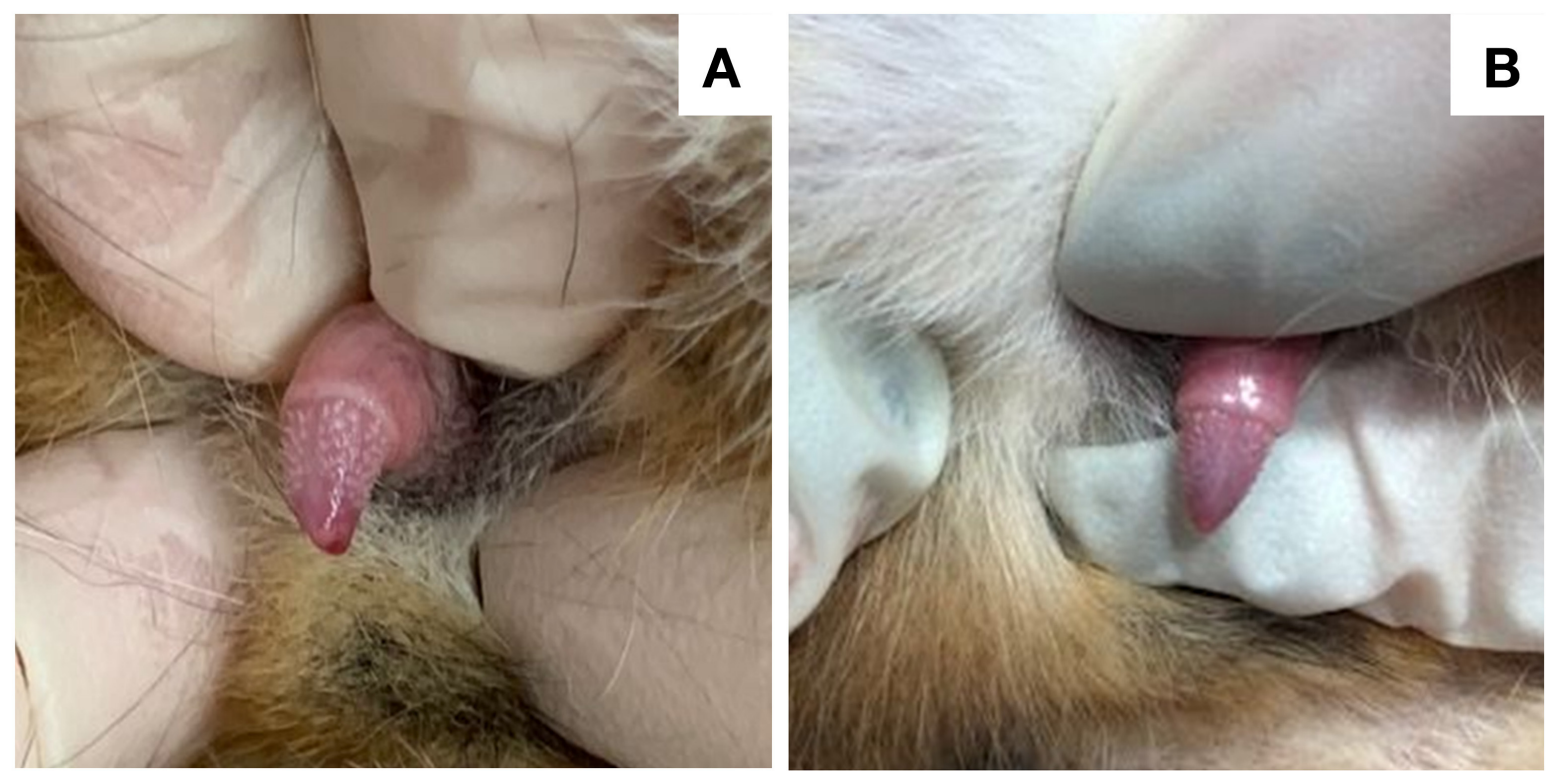
Images of the penis in a 7-year-old neutered male domestic shorthair cat (case 1) that underwent surgical adrenalectomy for a androstenedione-secreting adrenal tumor. Serum androstenedione concentration prior to surgery was 3.2 nmol/L (RI 0.35–2.1 nmol/L), which decreased to 0.35 nmol/L 3 weeks after surgery. **(A)** Penile barbs are apparent immediately prior to surgery. **(B)** The penile barbs had substantially regressed 2 weeks post-adrenalectomy.

Physical examination identified penile barbs in all male cats (Figure 1), and an enlarged clitoris in one of the three female cats (case 3). Other physical examination findings included a large and wide head (cases 1 and 4) and parasternal heart murmur (cases 2 and 8). One female cat (case 6) had a normal physical examination. One cat (case 2) was noted to have a firm, moderately distended, and non-expressible bladder on physical examination and was diagnosed with a lower urinary tract obstruction. This cat had no prior history of urinary tract obstruction. Signalment, owner concerns, and physical examination findings are summarized in Table 1.

**Table 1:** Summary of signalment, owner concerns, duration of clinical signs, and physical examination findings for 8 cats with androgen-secreting adrenal tumors.

	**Signalment**	**Owner concerns**	**Duration of signs**	**Physical exam**
Case 1	7 years M/C	Periuria Malodorous urine Vocalization Weight loss Hyperactivity	18 months	Penile barbs Large, wide head
Case 2	7 years M/C	Periuria Malodorous urine Vocalization Weight loss Aggression Polyphagia	1 year	Penile barbs Parasternal heart murmur Bladder distension
Case 3	7 yearsr F/S	Periuria Malodorous urine Aggression	1 year	Enlarged clitoris
Case 4	10 years F/S	Periuria Malodorous urine Vocalization Weight loss Vomiting	6 months	Large, wide head
Case 5	8 years M/C	Periuria Malodorous urine	>3 months	Penile barbs
Case 6	15 years F/S	Periuria Malodorous urine Vocalization	2 months	Normal
Case 7	11 years M/C	Periuria Malodorous urine Weight loss	3 days	Penile barbs
Case 8	9 years M/C	Aggression	1 week	Penile barbs Parasternal heart murmur

### 2.2. Clinicopathologic features

Routine laboratory testing, including a complete blood count and serum chemistry profile, was performed within 1 month of documenting increased androgen concentrations in five of the eight cats (cases 1, 2, 4, 7, and 8). None of these cats had increased ALT or ALP activities, hypercholesterolemia, or blood glucose concentrations >200 mg/dl. Similarly, serum sodium and potassium concentrations were normal in all five cats. The median potassium concentration was 4.4 mmol/L (range 4.0–4.8 mmol/L). The one cat with evidence of urinary obstruction on physical examination was azotemic (creatinine 10.2 mg/dl, reference interval 0.9–2.1 mg/dl). The azotemia resolved within 48 h of urinary catheterization and treatment with intravenous fluid. A urinalysis was performed in all seven cats that presented for inappropriate urination. Urine specific gravities ranged from 1.017–1.041. A sediment examination (*n* = 6) revealed no abnormalities except hematuria and pyuria in the one cat (case 2) with a urethral obstruction. Urine culture was negative for microbial growth in this cat.

Serum testosterone concentrations were measured in all cats, which was prompted by the 112 documentation of penile barbs or an enlarged clitoris in six of the eight cats (cases 1, 2, 3, 5, 7, and 8). Serum testosterone concentrations were high in seven of eight cats (cases 2–8), with a median concentration of 3.7 nmol/L (range 2.7–6.3 nmol/L, reference interval <1.2 nmol/L). In case 1, testosterone concentration was normal, but serum androstenedione concentration was increased 3.2 nmol/L (reference interval 0.35–2.1 nmol/L). One cat with high serum testosterone concentration (case 2) also had a high serum androstenedione concentration (3.9 nmol/L). Other corticosteroids were measured in five cats, including variable assessments of cortisol, aldosterone, progesterone, and estradiol. A summary of hormone concentration data and assay descriptions are available in Supplementary Table S1.

### 2.3. Diagnostic imaging

Abdominal ultrasound examination was performed in 7 cats (cases 1, 2, and 4–8), and either a right adrenal tumor (cases 1 and 6) or left adrenal tumor (cases 2, 4, 7, and 8) was identified in six of seven cases. The only cat in which adrenal enlargement was not observed during ultrasound examination had an adrenal nodule identified at the time of surgical exploration (case 5). Sonographic evidence of vascular invasion or metastatic disease was not observed in any cat. The contralateral adrenal gland ranged from 2.4 to 4.5 mm in dorsoventral thickness, which was considered normal in all cases (31). Uroliths were not observed in any cat. A computed tomographic examination also was performed in three cats (cases 1, 2, and 8) and confirmed the ultrasound findings in each case. The right adrenal lesion measured 1.2 cm × 1.2 cm × 1.3 cm in case 1. The caudal vena cava was mildly compressed dorsoventrally, but no filling defects were noted. Additionally, the prostate and bulbourethral glands were mildly enlarged in this cat. The left adrenal lesion measured 2.1 cm × 1.7 cm × 1.3 cm in case 2 and was causing medial deviation of the caudal vena cava and dorsal deviation of the aorta. No filling defects were noted although the phrenicoabdominal vein was poorly delineated in the region of the mass. In case 8, the left adrenal lesion measured 0.8 cm × 0.6 cm × 1.3 cm with no evidence of vascular invasion.

### 2.4. Treatment and outcomes

Surgical adrenalectomy was performed without complications in 4 cats (cases 1, 2, 5, and 8), all of which were discharged from the hospital 24–72 h after adrenalectomy. In case 1, periuria resolved several days later, and by 3 weeks, excessive night-time vocalization and hyperactivity had resolved. The cat was reevaluated at 3 weeks, and at this time, penile barbs had substantially regressed (Figure 1) and serum androstenedione concentration was 0.35 nmol/L (reference interval 0.35–2.1 nmol/L). The cat was alive and clinically normal >18 months after adrenalectomy. In case 2, periuria and malodorous urine resolved within 2 weeks, and excessive vocalization resolved 8 weeks post-adrenalectomy. At 2 and 8 weeks post-adrenalectomy, serum testosterone concentrations were <0.5 nmol/L (reference interval <0.5 nmol/L) on both occasions. The cat remained alive and clinically normal >1 year after adrenalectomy. In case 5, periuria and malodorous urine resolved 2 weeks after adrenalectomy, and the serum testosterone concentration was <0.3 nmol/L (reference interval <1.7 nmol/L) 4 weeks after surgery. The cat remained clinically normal for nearly 2 years, at which time a recurrence of urine marking and malodorous urine were reported. The serum testosterone concentration was again high at 3.1 nmol/L, and an abdominal ultrasound identified a mildly enlarged right adrenal gland (6 mm dorsoventral thickness). The owners declined additional diagnostics or treatments because of financial concerns. The cat remained alive, but symptomatic, 4 months after recurrence of clinical signs. In case 8, aggression persisted at 2 weeks and partially improved at 6 weeks post-adrenalectomy. At this time, penile barbs had substantially regressed, and serum testosterone concentration was normal (<0.7 nmol/L; reference interval, <1.2 nmol/L). Over the ensuing months, aggression fully resolved. The cat remained alive and clinically normal 16 months after adrenalectomy.

Medical management was pursued in three cats (cases 4, 6, and 7). Case 4 was treated with increasing doses of alprazolam (0.05 mg/kg PO q 24 h) which significantly improved, but did not resolve, the excessive vocalization. At 2 months after diagnosis of the adrenal tumor, megestrol acetate was prescribed (5 mg once per week) to help control newly developed roaming behaviors, but this cat was subsequently lost to follow up. Treatment with trilostane (8 mg/kg/day) was initiated in case 6. After 8 months of treatment, the testosterone concentration remained high at 16.7 nmol/L (reference interval <1.2 nmol/L), and a baseline cortisol concentration was 105 nmol/L (reference interval 15–97 nmol/L). Periuria, malodorous urine, and excessive vocalization persisted. The trilostane dosage was gradually increased (13 mg/kg/day). Partial improvement of periuria was reported, but no effects were observed on serum testosterone concentrations, which remained high at 20.6 nmol/L ~2 years after initial diagnosis. An ACTH stimulation test at this time revealed cortisol concentrations of 45 nmol/L and 40 nmol/L pre- and post-ACTH stimulation, respectively. The cat was euthanized nearly 3 years after diagnosis because of suspected intestinal neoplasia. In case 7, fluoxetine treatment (0.8 mg/kg PO q 24h) was initiated at the time of diagnosis, but clinical signs were unchanged. The cat remained alive 2 months after initial diagnosis.

No treatment was attempted in case 3, and the cat was euthanized. Necropsy examination revealed enlargement of the vulva and clitoris. There was no evidence of remnant gonadal tissue within the abdominal cavity. The right adrenal gland was round and enlarged measuring 1.2 cm × 0.9 cm × 0.9 cm. No gross metastases were observed.

### 2.5. Adrenal histopathology

**Figure 2:**
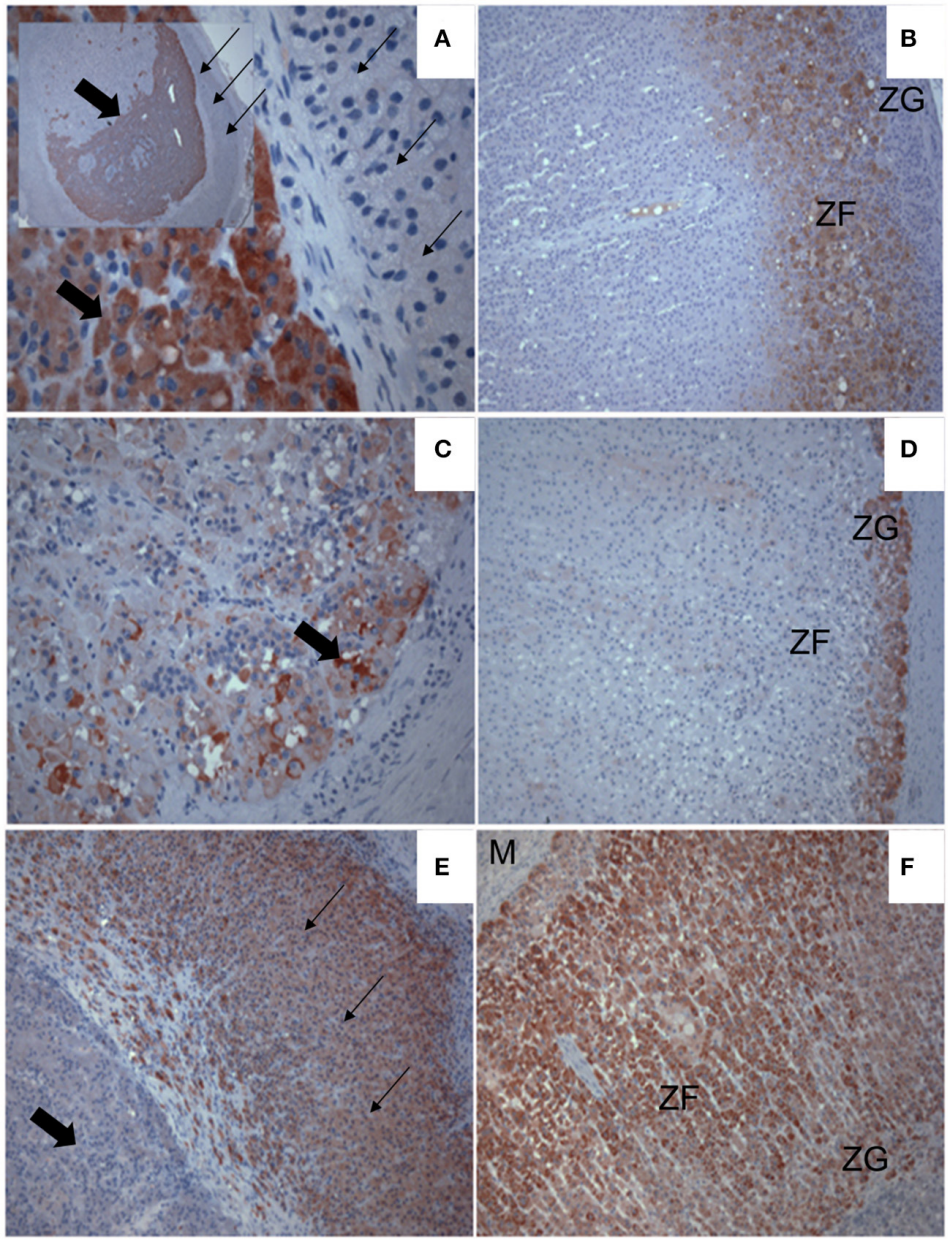
Immunohistochemical labeling showing expression (red chromagen deposit) of steroidogenic enzymes in an androgen-secreting adrenal mass **(A, C, E)** of a cat, and in normal feline adrenal cortex **(B, D, F)**. The expression of 17α-hydroxylase cytochrome P450 [P450c17; **(A, B)**], cytochrome b5 [b5; **(C, D)**] and cytochrome P450 21-hydroxylase [P450c21; **(E, F)**] are shown. **(A)** The inset shows the entire mass, surrounded by a compressed band of normal cortex (small arrows). Note P450c17 is strongly expressed in the developing tumor (block arrows) but is undetectable in the cortex around it (small arrows). **(B)** P450c17 expression is localized in the zona fasciculata (ZF) but not the glomerulosa (ZG) of a normal feline adrenal cortex. **(C)** Note b5 expression in lipid-laden tumor cells at the periphery of the mass. **(D)** Expression of b5 is detected in the ZG of normal feline adrenal cortex. **(E)** Expression of P450c21 is strong throughout the compressed cortex (small arrows) surrounding the tumor, which lacks detectable P450c21 expression by comparison (block arrow). **(F)** Note, the expression of P450c21 also extends throughout all cortical zones to the medulla (M) of the normal feline adrenal cortex.

**Figure 3:**
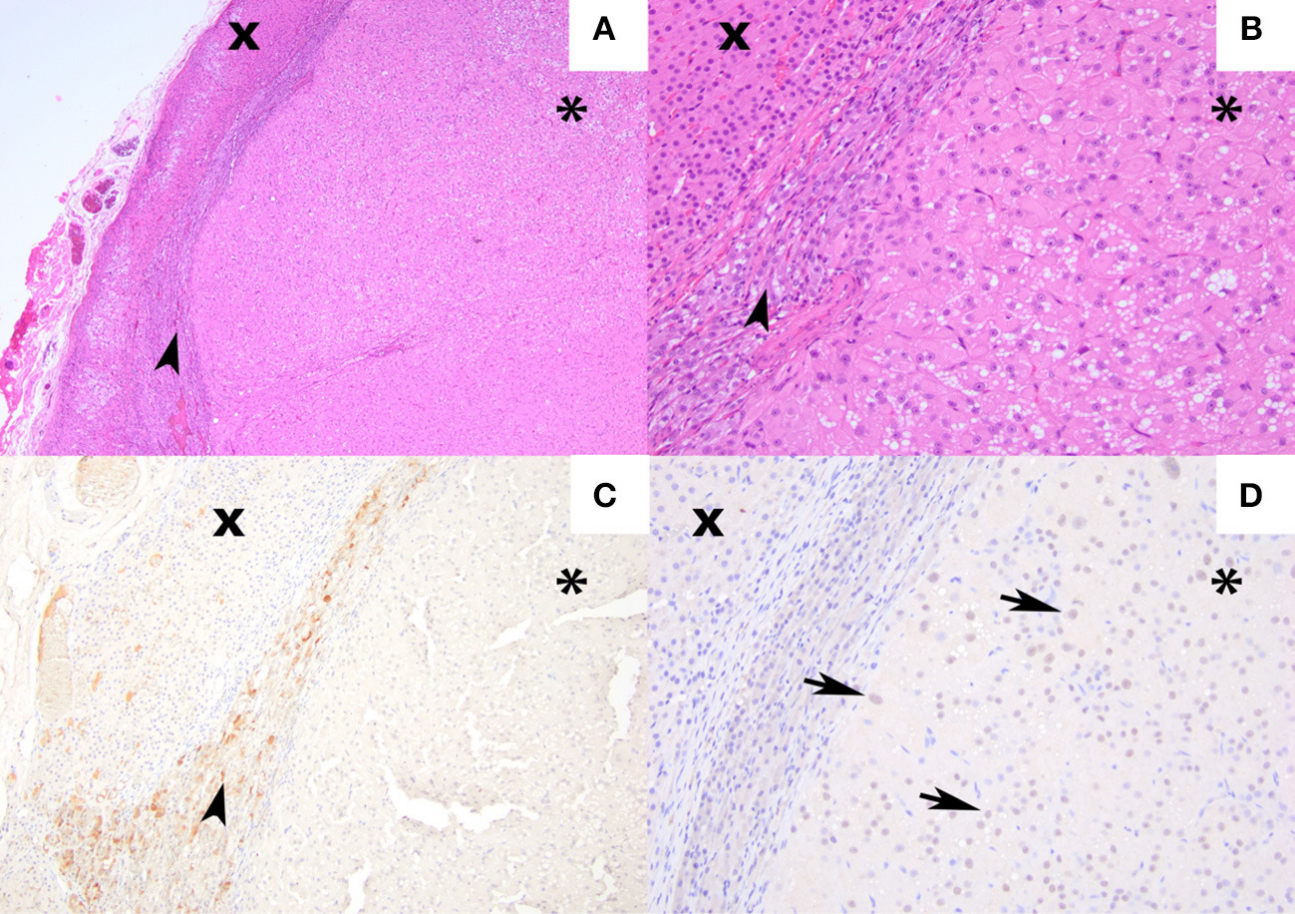
Hematoxylin and eosin staining and immunohistochemical labeling for met-enkephalin and GATA-4 from an androgen-secreting adrenal mass in a cat. **(A)** [4×] Hematoxylin and eosin stain of adrenocortical carcinoma (asterisk) compressing the adrenal medulla (arrowhead), and non-neoplastic adrenal cortex (×) **(B)** 100× magnification of the same region. **(C)** [10×] Medullary chromaffin cells expressing met-enkephalin. The neoplastic cells and non-neoplastic adrenal cortex are negative. **(D)** [20×] Approximately 60% of the neoplastic cells exhibit nuclear labeling for GATA-4 (arrows). The medullary chromaffin cells and non-neoplastic adrenal cortical cells are negative.

Adrenal tissue was available for histologic evaluation in five cats. Three cats (cases 2, 3, and 5) were diagnosed with an adrenocortical adenoma. The adenomas were composed of nodular to multilobular moderately cellular proliferations of neoplastic cells that formed broad trabeculae and sheets that compressed the adjacent medulla and normal adrenal cortex. Neoplastic cells were often plump, polygonal, and had large volumes of eosinophilic cytoplasm that often contained microvacuoles. Anisocytosis and anisokaryosis were mild with low numbers of mitoses (Figure 2). Although the neoplastic cells in the other 2 cats (cases 1 and 8) were similar in appearance, they either exhibited marked cellular atypia or extended into the capsule thus warranting a designation of carcinoma (Figure 3). To further characterize tumor etiology, immunohistochemical assessments were performed in three cases using antibodies and techniques described elsewhere (32–35). In one cat (case 3, Figure 2), the neoplastic cells exhibited strong immunoreactivity for 17 alpha-hydroxylase cytochrome P450 (P450c17) and cytochrome b5 (b5), but were negative for 21-hydroxylase cytochrome P450 (P450c21). In two cats (cases 1 and 8), neoplastic cells were negative for beta-endorphin and met-enkephalin, but exhibited strong nuclear labeling for GATA-4 (Figure 3).

## 3. Discussion

Herein, we describe eight cats with testosterone- or androstenedione-secreting adrenocortical tumors. All cats were initially evaluated for behavioral disorders including inappropriate urination, aggression, and excessive vocalization, alone or in combination. The majority of these cats had been empirically treated for lower urinary tract disorders or non-medical behavioral disorders with either incomplete or minimal response to therapy. Notable features included the commonly reported malodorous urine despite a normal urinalysis as well as the identification of masculinizing features in sexually altered cats. More specifically, malodorous urine was reported by seven of eight owners, and the identification of penile barbs or an enlarged clitoris prompted measurements of serum androgen concentrations in six of the eight cats. However, some of these historical and physical features were likely overlooked during early examinations in many cases. This could have been influenced by various factors such as the suspicion for more common conditions or perhaps the difficulty of evaluating the penis in a non-sedated cat. Regardless, these cases emphasize the importance of considering underlying medical pathology in cases of feline behavioral disorders, especially those that fail to respond empiric management strategies. They also underscore the importance of integrating historical information with a detailed physical examination.

Most functional adrenal tumors in cats are adenomas or adenocarcinomas arising from the zona glomerulosa that cause the clinical syndrome of primary hyperaldosteronism (16). However, adrenal cortical tumors can arise from any of the three layers of the adrenal cortex thereby resulting in various hormone abnormalities and associated clinical syndromes (17–25, 36). Adrenal androgenization is rare in cats though, and several alternative possibilities for androgen exposure were considered. No cats had evidence of gonadal tissue on physical examination or imaging studies. Exogenous exposure also was not reported and would seemingly be unlikely given the chronicity of clinical signs and identifiable adrenal lesions. Neoplastic adrenal tissue was confirmed by histologic evaluation in five cases, with either an adrenocortical adenoma or an adrenocortical adenocarcinoma diagnosed in each case. In the aggregate, the histologic assessments, lack of historical exposure to androgens, and absence of identifiable gonadal tissue provided strong support that the adrenal tumors were responsible for increased serum androgen concentrations in these cats.

Adrenal corticosteroid production is a complex and incompletely understood process, and current understanding of the numerous enzymatic pathways continues to evolve (37). Although androgen biosynthetic pathways have not been studied in detail in feline adrenal glands, we sought to further interrogate tumor etiology in several cases through immunohistochemical assessments of enzymes and transcription factors that are deemed critical in adrenal androgen production across species. The P450c17 enzyme is required for both androgen and cortisol synthesis whereas P450c21 is a key enzyme essential for cortisol synthesis. When co-expressed with P450c17, b5 augments the 17,20-lyase activity that leads to androgen rather than glucocorticoid synthesis (38–41). GATA-4 is a transcription factor involved in sex steroid synthesis and can be expressed in adrenocortical tumors, and its expression in adrenal tumors is best described in gonadectomized ferrets with sex-hormone producing adrenocortical tumors (33, 42, 43). The positive immunohistochemical assessments for P450c17 and cytochrome b5 coupled with the lack of labeling for P450c21 in the neoplastic tissue in one cat as well as the positive immunohistochemical assessments for GATA-4 in the neoplastic tissue in two cats provided strong support that the neoplastic cells were directly responsible for androgen secretion.

Adrenal cortical tumors are thought to account for <5% of pure androgen-secreting tumors in people, with most cases of testosterone secreting tumors attributed to ovarian tumors such as arrhenoblastomas or theca cell tumors (44, 45). In people, androgen-secreting adrenal tumors have been shown to secrete various androgens including dehydroepiandrosterone/dehydroepiandrosterone sulfate, androstenedione, or testosterone, with direct testosterone secretion being relatively uncommon (44, 45). Androstenedione concentrations were only measured in two cats, and dehydroepiandrosterone and dehydroepiandrosterone sulfate were not assessed in any cats. It is unknown if the high testosterone concentrations in the cats in our series were a direct result of adrenal secretion or if peripheral conversion of other adrenal androgens to testosterone could have occurred in some cases. Additional hormonal and immunohistochemical assessments would be needed to address this possibility, although the clinical relevance is questionable. Interestingly, testosterone secreting adrenocortical tumors in humans are most common in children and women (45–48), and all cats in our case series were neutered. Similarly, cats in previous reports of androgen-secreting adrenal tumors were neutered (26–30). This observation raises concern that the absence of androgen secretion from gonadal tissue could be a risk factor for the future development of androgen-secreting adrenal tumors. Further studies are needed to determine if there is an association of neuter status and sex-hormone secreting adrenal tumors in cats and to investigate potential underlying mechanisms for this phenomenon.

Surgical adrenalectomy is generally recommended for the treatment of adrenal tumors without overt evidence of metastatic disease (16). Indeed, the four cats that underwent adrenalectomy experienced improvement or resolution of clinical signs and long-term survival, which is consistent with clinical outcomes described in several previous case reports (26–30). However, adrenalectomy is invasive, expensive and unlikely to be pursued in all cases. Unlike medical treatment of hyperaldosteronism, which is usually associated with substantial clinical improvement and survival times >6 months, the medical treatment of androgen-secreting adrenal tumors in this series resulted in marginal success. This is not surprising as commonly used treatments for urine marking and aggression do not target androgen production or activity. Similarly, trilostane treatment did not alter clinical signs or testosterone concentrations in one cat in our series. However, trilostane treatment was previously reported to transiently improve urine marking and aggression in a cat with an estradiol- and testosterone-secreting adrenal tumor (49). Reasons for this discrepancy are not known, but the clinical improvement was not accompanied by improvements in serum androgen concentrations. Perhaps non-selective targeting of the adrenal cortex with mitotane would lower androgen concentrations, but mitotone is often poorly tolerated in cats (50). In people, androgen receptor blockers are available and commonly used to treat certain forms of prostate cancer (51). The use of these or similar classes of drugs would be intriguing in cases of androgen-secreting adrenal tumors in cats, but their use has not been reported in this species.

## 4. Conclusion

Androgen-secreting adrenal tumors might be an underrecognized cause of behavioral abnormalities in cats. Practitioners should maintain awareness of androgenization resulting from functional adrenal tumors as a differential diagnosis in neutered male and female cats with unexplained urine marking or aggression that are poorly responsive to treatment. A detailed history and physical examination are critical for clinical recognition, and the diagnosis is further supported by sonographic evaluation of the adrenal glands. Surgical adrenalectomy appears to be associated with a favorable outcome in most cases whereas medical management is likely to have limited effects.

## Data availability statement

The original contributions presented in the study are included in the article/Supplementary material, further inquiries can be directed to the corresponding author.

## Ethics statement

Ethical review and approval was not required for the animal study because this was a retrospective case series and not subject to IACUC review. However, the MSU CVM research committee reviewed this work. Written informed consent for participation was not obtained from cat owners because of the retrospective nature of this study.

## Author contributions

AC, CG, KB, JF, and DL were involved in clinical case management. KR assisted in case identification and endocrinology testing and interpretation. AG, AC, and DS assisted in histopathologic assessments. CE, AG, AC, KB, and DL wrote the original draft of the manuscript. All authors read, edited, and approved the final manuscript.
