# Supplementary material for: Case report: Androgen-secreting adrenocortical tumors in eight cats
Source: Front Vet Sci. 2023 Jun 13;10:1158142. doi: 10.3389/fvets.2023.1158142 (PMC10293747; doi:10.3389/fvets.2023.1158142)
Supplement: Supplementary file 1 [file Data_Sheet_1.docx]

**Table S1:** Summary of hormone concentration data for 8 cats with androgen-secreting adrenal tumors.

|  |  | Cat 1 | Cat 2 | Cat 3 | Cat 4 | Cat 5 | Cat 6 | Cat 7 | Cat 8 |
| --- | --- | --- | --- | --- | --- | --- | --- | --- | --- |
|  | RI | 7y M/C | 7y M/C | 7y F/S | 10y F/S | 8y M/C | 15y F/S | 11y M/C | 9y M/C |
| Aldosterone (pmol/L) | 194-388 |  |  |  |  |  |  | 159 | 216 |
| Andro (nmol/L) | 0.35-2.1 | 3.2 | 3.9 |  |  |  |  |  |  |
| Cortisol (nmol/L) | 15-97 | 66 | 66 |  |  |  |  | 154 | 159 |
| Estradiol (pmol/L) | 205-271 |  | 183 | 204 |  |  |  |  |  |
| Progesterone (nmol/L) | < 3.0 |  | 2.7 | 0.7 |  |  |  | 1.8 |  |
| Testosterone (nmol/L) | < 1.2 | < 0.7 | 2.7 | 3.7 | 6.3 | 3.5 | 5 | 3.7 | 3.2 |

All data in the table reflect baseline hormone concentrations. Androstenedione (Andro) concentrations were high in both cats (cases 1 and 2) in which this hormone was assessed. Two cats (cases 1 and 8) underwent complete ACTH stimulation testing, and post-ACTH stimulated (reference interval [RI], 97-207 nmol/L) cortisol concentrations in these 2 cats were normal at 139 nmol/L (case 1) and 159 nmol/L (case 8). One cat (case 2) underwent dexamethasone suppression testing, and cortisol concentration in this cat were < 27.5 nmol/L at both 4 and 8 hours after the administration of 0.1 mg/kg dexamethasone IV. More detailed assay descriptions can be found in the Appendix at the end of this document.

**Appendix: Corticosteroid Assay Descriptions.**

All samples for adrenal corticosteroids measurements were analyzed by American Association of Veterinary Laboratory Diagnosticians (AAVLD) accredited laboratories. Serum testosterone concentrations in 4 cats (cases 1, 4, 7, and 8) were measured at the Michigan State University Veterinary Diagnostic Laboratory (MSU-VDL) using a commercially available competitive chemiluminescent immunoassay (Immulite 2000 Testosterone, Siemens Healthcare Diagnostics, Gwynedd, UK). The manufacturer reports 0.6% cross-reactivity with androstenedione, 0.5% cross-reactivity with 5 α-Androstan-3β, 17β-diol, 2% cross-reactivity with 5α-Dihydrotestosterone, 0.7% cross-reactivity with methyltestosterone, and 0.1% cross-reactivity with progesterone. The manufacturer reports an analytical sensitivity of 0.5 nmol/L. Additional performance metrics were assessed at the MSU-VDL. Aliquots of feline serum samples of 0.4 and 15.3 nmol/L were mixed at volume combinations of 9:1, 3:1, 1:1, 1:3, and 1:9 and ran as samples in an assay. Recovery rates, expressed as % observed/expected, for the combinations were 89%, 100%, 109%, 98%, and 98% respectively. Assay repeatability was assessed with 3 pools of feline serum concentrations of 0.7, 4.4, and 26.8 nmol/L. The respective intraassay % coefficients of variation (CV) for 10 replicates of these pools were 20%, 9%, and 5%. Inter-assay repeatability %CVs for feline serum pools with mean testosterone concentrations of 0.8, 6.0, and 26.6 nmol/L were 39%, 17%, and 10%, respectively (n=10 assays). The reference interval of < 1.2 nmol/L was derived from evaluation of over 200 serum specimens from neutered male and female cats between the ages of 1 and 8 years that were submitted to the MSU-VDL endocrinology section. Serum testosterone concentrations in 3 cats were measured by either the Endocrinology Service at the University of Tennessee College of Veterinary Medicine Diagnostic Laboratory Services (UTCVM-DLS, cases 2 and 3) or IDEXX Laboratories (case 5) using the same competitive chemiluminescent immunoassay described above. The upper-end of the reference interval for serum testosterone concentrations in neutered cats established in these laboratories is 0.7 nmol/L for females and 1.7 nmol/L for males. Serum testosterone concentrations in the remaining cat (case 6) were measured using a previously available radioimmunoassay kit (Coat-a-Count, Siemens Medical Solutions Diagnostics, Los Angeles, California, USA) that has been described elsewhere (1).

Serum androstenedione concentrations (cases 1 and 2) were measured at UTCVM-DLS using a commercially available radioimmunoassay kit (ImmuChem Double Antibody Androstenedione RIA Kit, MP Biomedicals, LLC, Orangeburg, New York), which has been previously described for use in captive felids (2). Serum estradiol concentrations were measured in 2 cats (cases 2 and 3) at UTCVM-DLS using a commercially available radioimmunoassay kit (ImmuChem Double Antibody Estradiol RIA Kit, MP Biomedicals, LLC, Orangeburg, New York) that was described in the same report (2).

Serum cortisol concentrations in in 3 cats (cases 1, 7, and 8) were measured at the MSU-VDL using a commercially available chemiluminescent immunoassay (Immulite 2000 Cortisol, Siemens Healthcare Diagnostics, Gwynedd, UK) that has been described for cortisol measurements in cats elsewhere (3). Cortisol concentrations in 1 cat (case 2) were measured using the same methodology, but at the UTCVM-DLS. Serum progesterone concentrations were measured in 1 cat (case 7) at the MSU-VDL using a commercially available competitive chemiluminescent immunoassay (Immulite 2000 Progesterone, Siemens Healthcare Diagnostics, Gwynedd, UK), which has been described for use in cats elsewhere (3). Progesterone concentrations in 2 cats (cases 2 and 3) were measured at the UTCVM-DLS using the same methodology. Serum aldosterone concentrations in 2 cats (cases 7 and 8) were measured using a commercially available radioimmunoassay kit (ACTIVE® Aldosterone RIA, Beckman Coulter, IMMUNOTECH s.r.o., Prague, Czech) that has been described in detail elsewhere (3).

References for Assay Descriptions

1. Levy JK, Miller LA, Crawford PC, Ritchey JW, Ross MK, Fagerstone KA. GnRH immunocontraception of male cats. *Theriogenology*. (2004) 62(6):1116-1130. doi: 10.1016/j.theriogenology.2003.12.025.
2. Fecteau KA, Giori L, Cushing A, Price JM, Zhu X. Comparison of steroid and thyroid hormone concentrations in blood serum andplasma of captive tigers. *J Vet Diagn Invest*. (2022) 34(3):547-551. doi: 10.1177/10406387221090538.
3. Harro CC, Refsal KR, Shaw N, Alston SM, Folger W, Gross C, Cousins MW, Monahan CF, Mazaki-Tovi M, Langlois DK. Retrospective study of aldosterone and progesterone secreting adrenal tumors in 10 cats. *J Vet Intern Med*. (2021) 35(5):2159-2166. doi: 10.1111/jvim.16256.
